# NH_4_OH Treatment for an Optimum Morphological Trade-off to Hydrothermal Ga-Doped n-ZnO/p-Si Heterostructure Characteristics

**DOI:** 10.3390/ma11010037

**Published:** 2017-12-27

**Authors:** Abu ul Hassan Sarwar Rana, Hyun-Seok Kim

**Affiliations:** Division of Electronics and Electrical Engineering, Dongguk University-Seoul, Seoul 04620, Korea; a.hassan.rana@gmail.com

**Keywords:** ZnO, nanorod, Ga, doping, heterostructure, optoelectronics, hydrothermal

## Abstract

Previous studies on Ga-doped ZnO nanorods (GZRs) have failed to address the change in GZR morphology with increased doping concentration. The morphology-change affects the GZR surface-to-volume ratio and the real essence of doping is not exploited for heterostructure optoelectronic characteristics. We present NH_4_OH treatment to provide an optimum morphological trade-off to n-GZR/p-Si heterostructure characteristics. The GZRs were grown via one of the most eminent and facile hydrothermal method with an increase in Ga concentration from 1% to 5%. The supplementary OH^−^ ion concentration was effectively controlled by the addition of an optimum amount of NH_4_OH to synchronize GZR aspect and surface-to-volume ratio. Hence, the probed results show only the effects of Ga-doping, rather than the changed morphology, on the optoelectronic characteristics of n-GZR/p-Si heterostructures. The doped nanostructures were characterized by scanning electron microscopy, energy dispersive X-ray spectroscopy, X-ray diffraction, photoluminescence, Hall-effect measurement, and Keithley 2410 measurement systems. GZRs had identical morphology and dimensions with a typical wurtzite phase. As the GZR carrier concentration increased, the PL response showed a blue shift because of Burstein-Moss effect. Also, the heterostructure current levels increased linearly with doping concentration. We believe that the presented GZRs with optimized morphology have great potential for field-effect transistors, light-emitting diodes, ultraviolet sensors, and laser diodes.

## 1. Introduction

Because of its direct bandgap of 3.37 eV and high exciton binding energy of 60 meV at room temperature, ZnO has become one of the most important semiconductors in recent decades. The ease of fabrication processes has allowed the researchers to fabricate plenty of one-dimensional ZnO nanoscale shapes such as nanorods (NRs), nanowires, nanotubes, nanoflowers, nanoparticles, nanobelts, and many more [1,2,3,4]. Due to its enticing properties and structure, it has shown great potential in the realm of optoelectronic devices such as solar cells, field effect transistors, sensors, light emitting diodes, UV sensors, and laser diodes [5,6,7,8,9]. Furthermore, its chemical properties, such as biocompatibility, non-toxicity, and chemical stability, are useful for applications in cosmetics, medicine, and catalysis [10,11,12].

It is well known that ZnO is n-type because of the presence of many intrinsic donor defects [13]. Notwithstanding, it is important to control the intrinsic carrier concentration for optoelectronic device applications. It is believed that the highly doped ZnO, with least resistivity, may replace indium tin oxide, which is on the verge of extinction, as a transparent electrode [14]. Hence, ZnO doping is inevitable to control the majority carrier density for optoelectronic device applications. For this reason, group III elements, such as In (M_W_ 114.82), Ga (M_W_ 69.73), and Al (M_W_ 26.98), have been considered as the most suitable candidates because of the presence of an extra electron in their outermost shell [15,16,17]. Ga, being highly soluble in ZnO and a similar atomic radii with Zn, is one of the finest elements to dope ZnO without compromising its optoelectronic structure. Methods used to dope ZnO with Ga include radio frequency magnetron sputtering, molecular-beam epitaxy, arc-discharge, sol-gel, thermal evaporation, spray pyrolysis, pulsed laser deposition, metal-organic chemical vapor deposition, and hydrothermal method [18,19,20,21,22,23,24,25,26]. Nonetheless, the optoelectronic character of the fabricated devices with all the sophisticated methods may ensure better results, but we preferred hydrothermal method because of its simplicity, low cost, and ease of use [27].

Although, Ga-doping has already been used to influence the ZnO electronic and optical structure [28,29]. But, instead of mere speculations, it was difficult to cite the real reason of change in gallium-doped ZnO nanorods (GZR) optical and electrical characteristics because of changed ZnO morphology. For example, Wang et al. reported a redshift in photo-luminescent (PL) high-intensity UV peak which was ascribed to the combined effect of GZR decreased diameter and increased doping concentration [28]. On the contrary, Park et al. witnessed an increase in GZR diameter and a blue shift of high-intensity PL UV peak with an increase in doping concentration [29]. Furthermore, not only the morphology but the growth mechanisms were antithetical to each other and the reasons were ought to be addressed. In this study, we introduce NH_4_OH treatment for an optimum trade-off to hydrothermal Ga-doped n-ZnO/p-Si heterostructure characteristics. The goal of the study is to synchronize the NR morphology and dimensions so as the change in NR optical and electrical characteristics be conceived because of doping rather than changed morphology. In this context, the properties of undoped ZnO nanorods (UZRs) were compared and contrasted with GZRs grown via NH_4_OH treatment and with the GZR properties reported in the previous studies [28,29]. The GZR morphology was optimized by effectively controlling OH^−^ ion provision to the solution via NH_4_OH decomposition. Hence, despite morphology-induced change in surface-to-volume ratio, only the effects of Ga-doping were realized for GZR optoelectronic devices. The GZRs were characterized for morphological, structural, optical, elemental, and electrical characteristics.

## 2. Results and Discussion

### 2.1. GZR Morphology Dependence on Doped and Undoped Seeds

**Figure 1:**
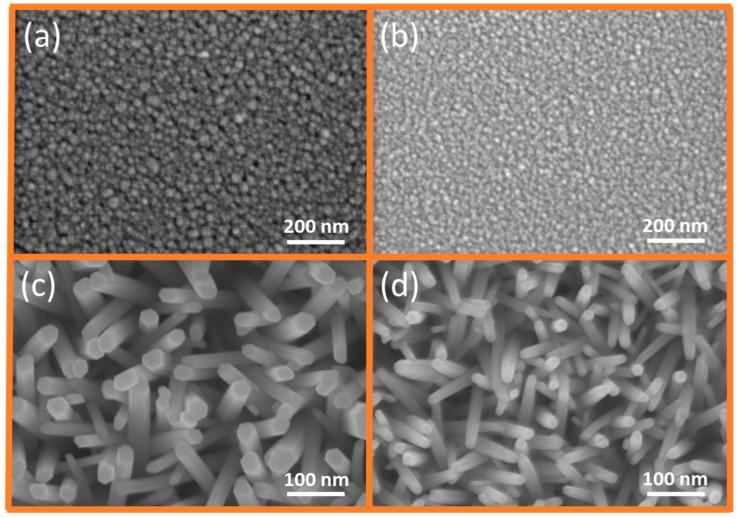
SEM images of: (**a**) undoped ZnO seeds; (**b**) Ga-doped ZnO seeds; (**c**) UZRs on undoped ZnO seeds; and (**d**) UZRs on Ga-doped ZnO seeds.

Figure 1 shows the plan-view scanning electron microscope (SEM) images of doped and undoped ZnO seeds and the UZR growth dependence upon seeds. It is already established that ZnO morphology and diameter depend upon seed particle size [30]. The doped and undoped seeds were used to monitor if there was any change in particle size of doped ZnO seeds. Instead of GZRs, only UZRs were grown on seeds to confirm the synchronized morphology change because of seeds and not because of Ga content in GZR growth solution. It is seen in Figure 1a,b that the particle size is shrunk in Ga-doped seeds. We believe that the shrunk morphology is because of the formation of Ga-Zn or Ga-OH clusters in seed solution. Similarly, the grown NRs on small diameter doped seeds have smaller dimensions than NRs grown on undoped seeds, as shown in Figure 1c,d. Furthermore, the vertical NR alignment confirms that the preferred orientation provided by Ga-doped seeds is along 0001 rather than 2**11**0 or 11**1**0 directions. Hence, throughout the experiments, we used Ga-doped ZnO seeds for GZR growth to minimize surface free energy between GZRs and Si substrates and to provide a smooth basic growth units to GZRs. Similarly, it is also substantiated that Ga-doped ZnO thin films can also be fabricated for thin-film-based solar cell applications.

### 2.2. GZR Morphology without NH_4_OH Treatment

**Figure 2:**
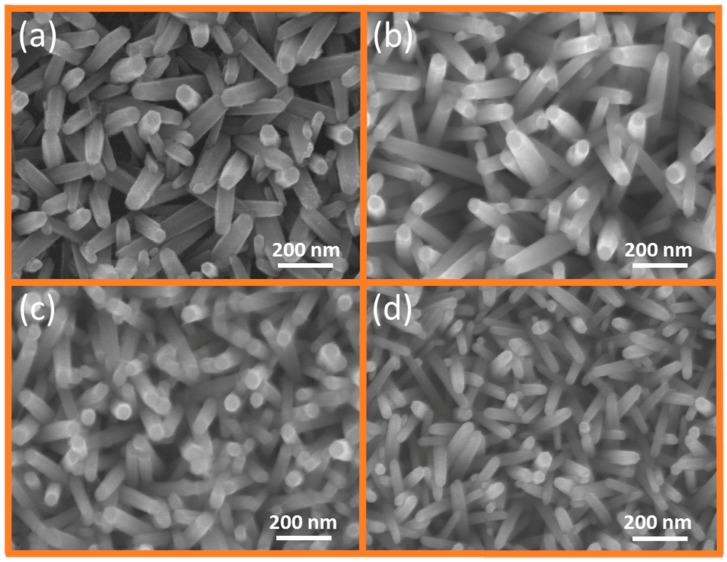
SEM images of: (**a**) UZRs; (**b**) 1% GZRs; (**c**) 2% GZRs; and (**d**) 5% GZRs.

To probe into the effects of NH_4_OH treatment, different doping concentration GZRs were first grown without the use of any surfactants or NH_4_OH. Figure 2a–d show the plan-view SEM images of UZRs, 1%, 2%, and 5% GZRs, respectively. In contrast to the findings of Park et al. our results support experimental results of Wang et al. [28,29]. In the absence of any additives, the Ga^3+^ reacts with OH^−^ ions in the solution provided by the decomposition of methenamine and supports homogeneous nucleation of reactants in the solution against heterogeneous nucleation on seeds. The regular OH^−^ ions supply is quite vital for NR growth and their shortage may result in morphological changes in general or decrease in NR diameter in particular. The UZRs have the largest diameter which keeps on decreasing as the doping level increases from 1% to 5%, which supports high homogeneous nucleation rates in the solution. Only a small change is seen in the diameters of UZRs and 1% and 2% GZRs, but a gross change in diameter is seen between UZRs and 5% GZRs. However, instead of the large diameter GZR lateral growth, the high concentration 5% GZRs are also oriented well along 0001 direction, which is in contrast to the findings of Wang et al. [28]. We believe that the axial growth of even a highly doped sample is because of Ga-doped seeds which support one of the highest growth rates in 0001 direction and render an additional benefit to GZR growth [31]. 

### 2.3. NH_4_OH Treatment for Optimum Morphological Trade-off

**Figure 3:**
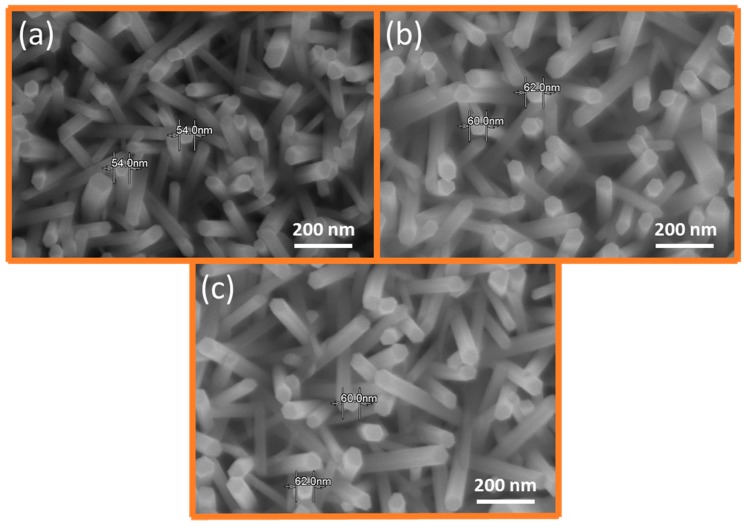
SEM images of: (**a**) 1%; (**b**) 2%; and (**c**) 5% GZRs grown with NH_4_OH treatment.

The longstanding controversy regarding a doping-centric change in ZNR morphology and its reasons and solutions are addressed via NH_4_OH treatment. The GZR morphology control is important because it affects the GZR surface-to-volume ratio and ultimately influences their optical and electrical properties [32]. Previously, it was difficult to substantiate either the change in optical and electrical properties was doping-centric or because of the morphology-induced change in GZR surface-to-volume ratio. Hence, we exploited NH_4_OH treatment to address the problem by optimizing GZR morphology, specifically GZR diameter, for different doping concentrations, and the phenomenon is called as GZR morphological trade-off. Figure 3 shows the SEM images of GZRs grown with NH_4_OH treatment. The idea was to control the morphology by an optimum provision of OH^−^ ions via NH_4_OH decomposition in the solution. The additional OH^−^ ions impart a trade-off for OH^−^ ions that were wasted in Ga-OH complex formation and homogeneous nucleation. Hence, the average diameter of 1%, 2%, and 5% GZRs remained fixed at ~60 nm, as shown in Figure 3a–c, respectively. For best results, the amount of NH_4_OH ought to be controlled judiciously because a slight increase or decrease may alter results. In this study, we used 5, 7, and 10 mL NH_4_OH for 1%, 2%, and 5% GZRs, respectively.

### 2.4. GZR Isoelectric Point-Dependent Growth Mechanism

**Figure 4:**
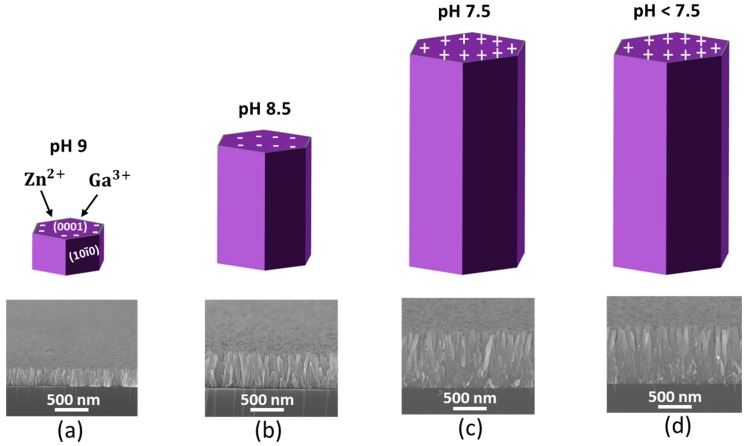
GZR isoelectric point-dependent growth mechanism with their corresponding cross-sectional SEM images at (**a**) 30 min; (**b**) 2 h; (**c**) 4 h; and (**d**) 6 h.

The GZR growth mechanism is better explained by isoelectric point-centric surface charge reversal phenomenon, as shown in Figure 4. The ZnO isoelectric point, without the addition of any surfactant, ranges from 7.4 to 8.2 [33]. ZnO nucleation and growth depend upon pH-centric surface charge. Initially, the solution pH was 7 and no nucleation was promoted. With methenamine decomposition, the solution pH was naturally raised to 9 and 0001 surface charge was reversed from positive to negative, as shown in Figure 4a. As soon as the surface charge was reversed, the Zn^2+^ and Ga^3+^ ions were deposited on the negative 0001 and the O^−^ ions were deposited on the 000**1** positive surfaces. The heterogeneous nucleation process kept working similarly until pH was reduced to 7.5 because of OH^−^ ion extinction in the solution and the 0001 surface charge was reversed to positive. This particular point is called as growth stoppage point beyond which the growth is not promoted.

The said phenomenon was also substantiated with the help of cross-sectional SEM images of NRs on corresponding pH values in Figure 4. The NRs kept growing as the pH value decreased from its zenith at 9 and back to 7.5. Any rise in temperature or time beyond this point did not support nucleation, where NR length remained the same, as shown in Figure 4c,d. In the presence of Ga, the solution isoelectric point was shortly reached because of an utter wastage of OH^−^ ions via homogeneous nucleation in the form of Ga-OH complexes. Hence, GZR diameter was reduced because of an early surface charge reversal as the Ga concentration was increased in the solution. With the addition of NH_4_OH, the surface charge reversal duration and nucleation process were extended by raising the pH to the maximum value of 10.5 for 5% GZRs.

### 2.5. Elemental Characteristics of Ga-Doped Seeds and GZRs

**Figure 5:**
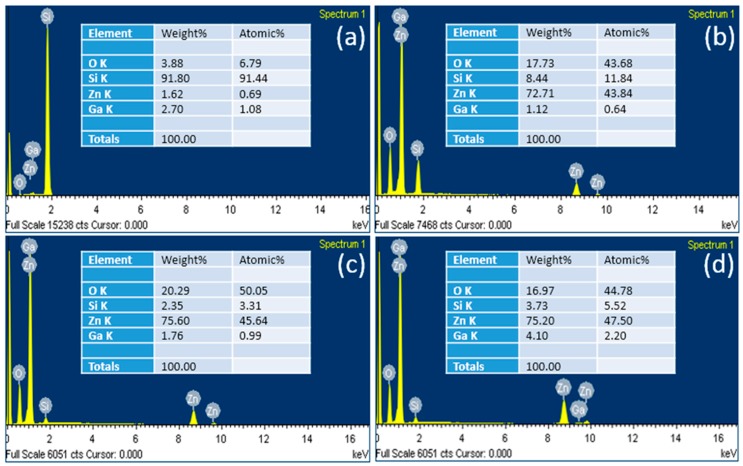
EDS of (**a**) Ga-doped ZnO seeds; and (**b**) 1%; (**c**) 2%; and (**d**) 5% GZRs grown with NH_4_OH treatment.

The elemental characteristics of Ga-doped ZnO seeds and GZRs grown with NH_4_OH treatment were measured with energy dispersive X-ray spectroscopy (EDS), as shown in Figure 5. The insets explain the detailed atomic and weight percentages of the found elements in a nanostructure. The found elements in all the samples were Zn, O, Si, and Ga. Figure 5a confirms the incorporation of Ga into ZnO seeds. The large Si peak is from the substrate because of a very thin layer of twice coated ZnO seeds. Similarly, all the GZR samples in Figure 5b–d have Ga peaks with an increased intensity as the doping level increases. The point to ponder is an increase in the Ga atomic percentage as the doping level increases from 1% to 5%. The highest number of Ga atoms are found in 5% GZRs, which confirms the effective incorporation of Ga even in high doping concentrations via our method. The sole purpose of EDS was to confirm the incorporation of Ga into ZnO lattice and the effectiveness of the doping method. With the presented EDS results, the presence of Ga ions in the host can be determined. However, in order to measure the proper stoichiometry of elements, the silicon substrate must not be taken into account in the calculation, which is present in all the EDS samples in Figure 5. Perhaps, we tried a lower EDS acceleration voltage to avoid excitations from the Si substrate but all went in vain and we found Si peaks in all quantifications. Hence, it is impossible to drive conclusions from the current EDS quantification regarding the stoichiometry and proper ratio of elements.

### 2.6. Structural Characteristics of GZRs

**Figure 6:**
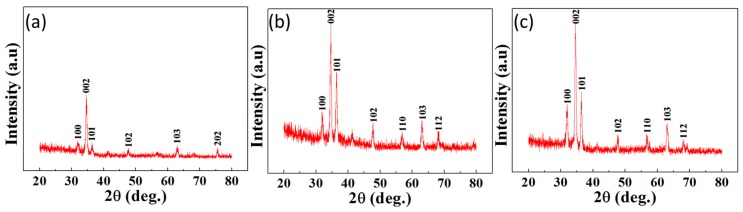
XRD of: (**a**) 1%; (**b**) 2%; and (**c**) 5% GZRs.

GZR structural characteristics were found with X-ray diffraction (XRD) crystallography, as shown in Figure 6. All the samples show a typical hexagonal wurtzite ZnO phase and none of the secondary diffraction phases, such as ZnGa_2_O_4_ and Ga_2_O_3_, are seen in the XRD response of all the samples [34]. Hence, despite Ga incorporation into ZnO crystal lattice, it is inferred that Ga-doping does not influence ZnO structural phase. The presence of multiple peaks along 100, 002, 101, 102, 110, 103, and 112 affirms the GZR polycrystalline nature in all samples. Despite different intensity XRD peaks, the highest peaks in all the samples are along 002 direction. The low intensity peaks along 100 and 101 certified a slight *a*-axis orientation in all the samples because of partly inclined GZRs. However, the 100 and 101 peak intensities are smaller and impotent as compared to 002 peak intensity, which confirms the *c*-axis orientation of GZRs in all the samples. Since the ionic radii of Ga^3+^ (0.06 nm) and Zn^2+^ (0.07 nm) are almost identical, large ZnO lattice distortions were already not expected, which was certified by an increase in 002 peak intensity with an increase in doping concentration [35]. The detailed lattice parameters are provided in Table 1. The lattice constant was calculated with Bragg’s law [36]. It is certified that the 2θ position of 002 peaks remains almost fixed for 1% and 2% GZRs except for heavily-doped 5% GZRs. Furthermore, a decrease in full-width of half maximum (FWHM) of 002 peak affirms the GZR improved crystallinity. Expecting a large stress/strain in GZR direct growth on bare Si substrate because of large lattice mismatch, GZRs were grown on a buffer layer of Ga-doped ZnO seeds. Hence, the extrinsic factors for stress can be eliminated and the intrinsic stress/stain along the GZR *c*-axis and in thin GZR film were calculated via XRD data analysis. The *c*-axis strain (ε_c_) is

(1) $$\varepsilon_{c}=\frac{c-c_{o}}{c_{o}}\times 100,$$

where c_o_ and c are the lattice constants of stress free bulk ZnO (0.5205 nm) and GZRs, respectively. Although Ga adds minimum lattice vibrations to GZRs, yet the heavily-doped sample shows the highest strain along *c*-axis (Table 1). The stress (σ) in GZRs can also be calculated via biaxial stress model [37],

(2) $$\sigma =\frac{2{\left(C_{13}\right)}^{2}-C_{33}\left(C_{11}+C_{12}\right)}{2C_{13}}\varepsilon ,$$

where $C_{11}$, $C_{12}$, $C_{13}$, and $C_{33}$ are the bulk ZnO elastic stiffness constants with value 208.8, 119.7, 104.2, and 213.8 GPa, repectively. Hence,

(3) σ=−233 ε.

The calculated stress/stain values are reported in Table 1, where the negative sign for strain shows that the strain is compressive for all the doped samples.

### 2.7. Optical Properties of GZRs

**Figure 7:**
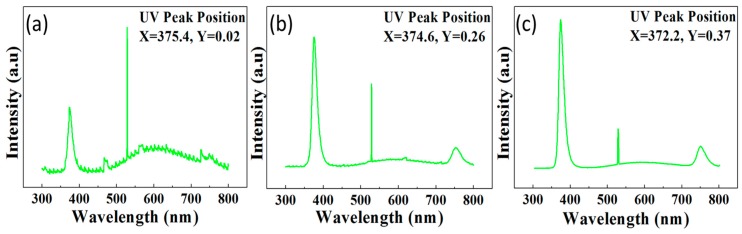
PL spectra of (**a**) 1%; (**b**) 2%; and (**c**) 5% GZRs.

Figure 7 depicts the room temperature PL spectra of GZRs in the range of 300 nm to 800 nm. The PL spectra show three distinct peaks in UV, visible, and near IR regions. The sharp and highest peak in UV region is a direct response of free exciton recombination via a near band edge exciton-exciton collision process [38]. The UV peak positions on X and Y scales are highlighted in Figure 7 panels. It can be seen in the Figure 7 that the UV peak intensity increases with Ga-doping concentration which verifies the better optical characteristics of heavily doped 5% GZRs. Another point to ponder in this particular backdrop is a blue shift of UV peaks with an increase in doping concentration. There are four conflicting theories regarding GZR-assisted UV peak position shift which are of paramount importance. First, a redshift in UV peak position is reported with an increase in Ga concentration because of doping-induced band gap renormalization (BGR) effect [39]. An increase in carrier concentration results in free carrier screening via many-body interactions which influence BGR effect. Second, extrinsic and intrinsic stress and lattice distortions may also influence peak shift position [40]. Third, a morphology induced change in surface-to-volume ratio can have a grave impact upon a change in the peak position [41]. Forth, the peaks are blue shifted because of doping-induced Burstein-Moss (BM) effect [42]. Previously, it was difficult to trace the real reason of a change in optical characteristics because of changed morphology and stress centers in GZRs. In this study, we support the description given my BM effects because all the samples were blue shifted. Furthermore, the reduced dimeter-induced change in surface-to-volume ratio cannot be considered as the GZR diameter was synchronized to capture the real reason for this particular shift. It has also been reported in the structural characteristics that the grown GZRs were stress-free and no lattice distortions were monitored because of GZR growth on Ga-doped seeds. Hence, the samples were blue shifted because of BM effects, where the Fermi level was moved towards the conduction band as the doping concentration increased from 1% to 5%. The exciton recombination from an increased energy bandgap emits in lower wavelength regions.

Similarly, the GZR optical properties differ in the wavelength range of 500 to 700 nm. Generally, ZnO shows a broad and high peak in the visible band is believed to be a direct response to many Zn interstitial and oxygen vacancy defects [35]. Herein, the 1% GZRs show more or less a similar trend with UZRs but the peak moves toward a flat band condition with an increased Ga concentration. It is inferred that Ga atoms tend to replace the defect centers which respond in the visible region. An increase in UV peak intensity and a decrease in visible peak intensity with an increased doping concentration show that Ga doping improves the ZnO optical properties. Hence, Ga-doping is an important way to fix the naturally occurring defects and to improve the ZnO crystalline structure. Furthermore, it is also found that Ga-doping creates some defects which respond to near IR region as shown by the peaks around 750 nm.

### 2.8. Carrier Concentration and Electrical Characteristics of GZRs

**Figure 8:**
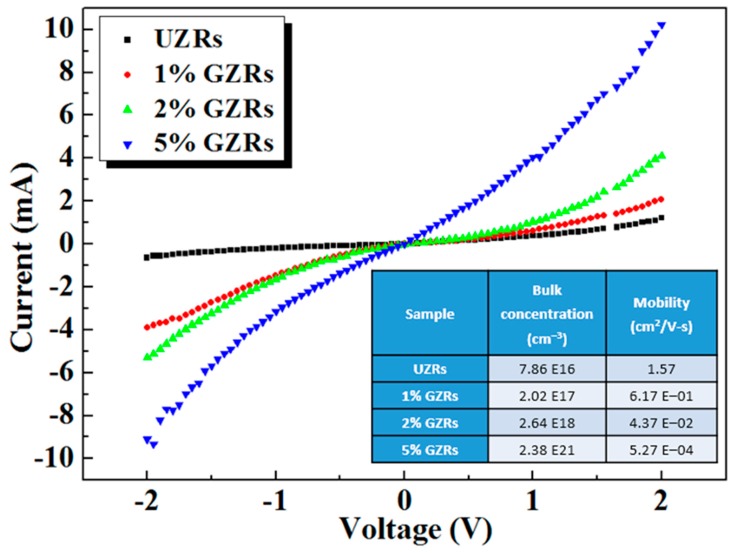
Electrical characteristics of UZRs and GZRs. Bottom inset table reveals the Hall-effect measurements.

Despite bandgap engineering and exciton energy, the optoelectronic device characteristics largely depends upon the GZR electronic character such as majority carrier concentration and carrier mobility. The GZR carrier concentration was found with a four-probe Hall-effect measurement system under dark conditions. For a smooth carrier transport during Hall-measurement, GZRs were grown on an insulating glass substrate and an ohmic-In contacts were made around the four corners of the grown GZR film. For better results, the GZR dimensions on glass substrate were synchronized with the dimensions on p-Si substrate. The detailed experimental findings are illustrated in inset table in Figure 8. It is found that Ga-doping increases majority carrier concentration. The hydrothermally grown UZRs already have high intrinsic carrier density because of the incorporation of many donor defects during growth. Because of having an extra electron in the outermost shell (4S^2^ 4P^1^), Ga-doing is an effective way to increase the average carrier concentration from 10^16^ in UZRs to 10^21^ in 5% GZRs.

**Figure 9:**
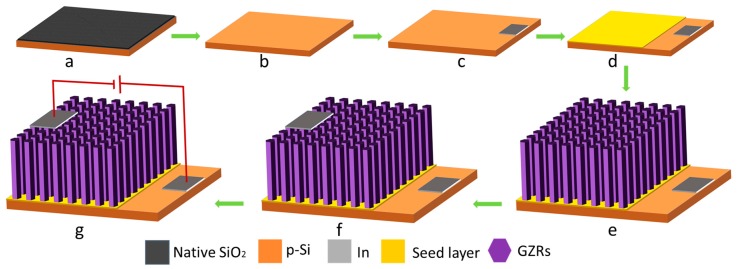
Device fabrication process flow diagram.

The current-voltage (I-V) response of UZRs and GZRs-based n-ZnO/p-Si heterostructures were found with Keithley 2410 in a probe station under dark conditions. The I-V response is shown in Figure 8 and the schematics of the heterostructure device with probe station contacts are shown in Figure 9g. It can be seen that all the samples show a diode-like I-V behavior forming an ohmic contact with In. The current density increases with an average increase in majority carrier concentration and the highest current levels are achieved in the highest doped 5% GZRs. The electrical characteristics of our samples are comparable with the electrical characteristics of sol-gel spin-coated Al-doped ZnO films [43]. It is affirmed that the electrical behavior is a carrier concentration-dependent direct response of GZRs rather than an increase or decrease in morphology-induced surface-to-volume ratio.

## 3. Materials and Methods

### 3.1. Substrate Cleaning, Bottom Electrode Deposition, and Seed Coating

To conduct all the experiments, commercially available chemicals were purchased from Sigma Aldrich. To make a p-n heterojunction, n-GZRs were grown on p-Si (100, 1–10 Ω·cm) substrates. Figure 9 shows the process flow diagram of the experimental procedures for device fabrication. Si reacts with oxygen in ambient conditions to form an insulating SiO_2_ layer on the surface, as shown in Figure 9a. Hence, Si substrates were immersed in buffered oxide etchant (BOE) (6:1) to remove the native SiO_2_ layer for a smooth working of a p-n junction device. After two min, the substrates were removed from BOE and cleaned with deionized (DI) water (18 MΩ) and N_2_ gas and a clean substrate was ready for device fabrication, as shown in Figure 9b. Next step was bottom In electrode deposition, which was done via photolithography and metal evaporation, as depicted in Figure 9c. Doped ZnO seeds were made by mixing gallium nitrate hydrate [Ga(NO_3_)_3_·xH_2_O] (M_W_ 255.74 anhydrous basis) and zinc acetate dihydrate [Zn(CH_3_COO)_2_·2H_2_O] (M_W_ 219.51 g/M) in n-propanol [C_3_H_8_O] (M_W_ 60.10 g/M). The chemicals were sonicated for 30–40 min for saturated seed solution. The Ga-doped seeds were spun twice on the substrate surface at 3000 RPM for 2 min. To provide seed stability, the spin-coated seeds were annealed at 100 and 300 °C on a hotplate for successive coatings. Finally, a thin layer of Ga-doped ZnO seeds was formed to assist vertical GZR growth, as shown in Figure 9d.

### 3.2. GZR Growth and Heterostructure Device Fabrication

The growth solution was made by mixing 25 mM each of zinc nitride hexahydrate [Zn(NO_3_)_2_·6H_2_O] (M_W_ 297.48 g/mol) and methenamine [C_6_H_12_N_4_] (M_W_ 140.186 g/mol) in DI water. For doping, three different growth solutions were prepared by adding 0.252, 0.510, and 1.315 mM gallium nitrate hydrate into the above solutions to fix the Ga-doping to 1%, 2%, and 5%, respectively. The doping molarity to percentage conversion was calculated by the relation: Ga% = M_Ga_/(M_Ga_ + M_Zn_) × 100%, where M_Zn_ and M_Ga_ are the respective molar concentrations of Zn and Ga. Also, different amounts of ammonium hydroxide [NH_4_OH] (M_W_ 35.05 g/M with 28% NH_3_ in H_2_O) were added into the solutions after intermittent intervals to provide GZR morphology trade-off. The growth solutions were subjected to a magnetic stirring for 1 h without heating. After 1 h, the seeded substrates were immersed upside down into solution autoclaves and was heated at 90 °C. After 4 h, the substrates were removed from the autoclaves, rinsed in DI water, and dried with N_2_ and vertical GZRs were grown on the seeded substrates, as shown in Figure 9e. The top in electrode was deposited directly upon GZRs with photolithography and metal evaporation in the opposite direction to the bottom In electrode, as depicted in Figure 9f. The probe station contacts for electrical measurements were made in a way portrayed in Figure 9g.

### 3.3. Material Characterizations

The photolithography and In contacts were made with Karl Suss MA-6 (Dongguk University, Seoul, Korea) and E-beam metal evaporator (Dongguk University, Seoul, Korea). The GZR morphology was seen with SEM (Hitachi S-4800, Suwon, Gyeonggi-do, Korea) operating at 25 KeV. Ga incorporation into ZnO and elemental proportions were confirmed with EDS attached to SEM equipment. The GZR structural characteristics were measured with XRD (Rigaku Ultima IV, Dongguk University, Seoul, Korea) from 2θ values of 20 to 80 degrees at λ = 1.5418 Å. The optical properties were monitored with PL spectroscopy (Accent RPM 2000, Suwon, Gyeonggi-do, Korea) from 300 nm to 800 nm at room temperature and pressure. The I-V diode characteristics were measured with Keithley 2410 in a probe station under the dark condition and the majority carrier concentration was found using Hall-effect measurement system (ECOPIA AHT55T5, Dongguk University, Seoul, Korea). The in-situ solution temperature and pH were monitored with a digital thermometer and pH meter, respectively. 

## 4. Conclusions

In this study, we present NH_4_OH treatment for an optimum morphological trade-off to hydrothermal n-GZR/p-Si heterostructures. NH_4_OH treatment was necessary to provide an additional OH^−^ ion supplement to synchronize GZR morphology for high doping levels. For best results, the supplementary OH^−^ ions were optimally tuned for different doping concentrations. Hence, rather than changed morphology induced characteristic transition, it was easy to probe the real essence of GZR optoelectronic characteristics, which was not the case in previous studies. In this context, the GZR morphological, elemental, structural, optical, and electrical properties were analyzed. The morphology of all the doped samples was tuned to an average diameter of ~60 nm. EDS results showed that Ga was incorporated into ZnO lattice and Ga atomic concentration increased with doping. The decreased lattice constant and increased 002 XRD peak intensity depicted that Ga-doping improved the crystalline ZnO structure without any lattice distortions. PL results showed that increased Ga concentration shifted the UV peak towards the lower wavelengths because of carrier concentration-induced BM effects, and Ga tend to fix the intrinsic ZnO defects and improve GZR optical characteristics. Furthermore, the GZR electrical characteristics were also improved by Ga-doping. The carrier concentration reached as high as 10^21^ cm^−3^ for 5% GZRs. All the samples showed a diode like I-V behavior with a uniform increase in current intensity with doping concentration. The propounded results may provide impetus to the study on optoelectronic characteristics of ZnO-based heterojunction devices.

## Figures and Tables

**Table 1:** Lattice parameters of GZRs.

Ga-Doping (%)	002 Position (Deg.)	a (Å)	c (Å)	c/a	FWHM (Rad.)	$\varepsilon_{c}$ (%)	σ (GPa)
1	34.38	3.25	5.20	1.60	0.005	−0.07	0.17
2	34.39	3.25	5.20	1.60	0.004	−0.03	0.08
5	34.60	3.24	5.18	1.59	0.002	−0.48	0.94

## References

[1] Shi Y., Bao S., Shi R., Huang C., Amini A., Wu Z., Zhang L., Wang N., Cheng C. (2016). Y-shaped ZnO Nanobelts Driven from Twinned Dislocations. Sci. Rep..

[2] Yang Z., Wang M., Shukla S., Zhu Y., Deng J., Ge H., Wang X., Xiong Q. (2015). Developing Seedless Growth of ZnO Micro/Nanowire Arrays towards ZnO/FeS_2_/CuI P-I-N Photodiode Application. Sci. Rep..

[3] Rana A.S., Kang M., Jeong E.S., Kim H.S. (2016). Transition between ZnO Nanorods and ZnO Nanotubes with Their Antithetical Properties. J. Nanosci. Nanotechnol..

[4] Tachikawa S., Noguchi A., Tsuge T., Hara M., Odawara O., Wada H. (2011). Optical Properties of ZnO Nanoparticles Capped with Polymers. Materials.

[5] Alenezi M.R., Henley S.J., Silva S.R.P. (2015). On-chip Fabrication of High Performance Nanostructured ZnO UV Detectors. Sci. Rep..

[6] Lee C.T. (2010). Fabrication Methods and Luminescent Properties of ZnO Materials for Light-Emitting Diodes. Materials.

[7] Kim K.H., Utashiro K., Abe Y., Kawamura M. (2014). Structural Properties of Zinc Oxide Nanorods Grown on Al-Doped Zinc Oxide Seed Layer and Their Applications in Dye-Sensitized Solar Cells. Materials.

[8] Kang M., Rana A.S., Jeong E.S., Kim H.S. (2017). Direct Observation of Thermally Generated Electron-Hole Pairs in ZnO Nanorods with Surface Acoustic Wave. J. Nanosci. Nanotechnol..

[9] Zong X., Zhu R. (2017). Zinc oxide nanorod field effect transistor for long-time cellular force measurement. Sci. Rep..

[10] Pineda-Hernandez G., Escobedo-Morales A., Pal U., Chigo-Anota E. (2012). Morphology evolution of hydrothermally grown ZnO nanostructures on gallium doping and their defect structures. Mater. Chem. Phys..

[11] Choi Y.E., Kwak J.W., Park J.W. (2010). Nanotechnology for Early Cancer Detection. Sensors.

[12] Chu D., Masuda Y., Ohji T., Kato K. (2010). Formation and Photocatalytic Application of ZnO Nanotubes Using Aqueous Solution. Langmuir.

[13] Wang Q., Yan Y., Zeng Y., Lu Y., Chen L., Jiang Y. (2016). Free-Standing Undoped ZnO Microtubes with Rich and Stable Shallow Acceptors. Sci. Rep..

[14] Sun D., Tan C., Tian X., Huang Y. (2017). Comparative Study on ZnO Monolayer Doped with Al, Ga and In Atoms as Transparent Electrodes. Materials.

[15] Koida T., Kaneko T., Shibata H. (2017). Carrier Compensation Induced by Thermal Annealing in Al-Doped ZnO Films. Materials.

[16] Babar A.R., Deshamukh P.R., Daekate R.J., Haranath D., Bhosale C.H., Rajpure K.Y. (2008). Gallium doping in transparent conductive ZnO thin films prepared by chemical spray pyrolysis. J. Phys. D Appl. Phys..

[17] Biswal R., Maldonado A., Vega-Perez J., Acosta D.R., Olvera D.L.L. (2014). Indium Doped Zinc Oxide Thin Films Deposited by Ultrasonic Chemical Spray Technique, Starting from Zinc Acetylacetonate and Indium Chloride. Materials.

[18] Tseng J.Y., Chen Y.T., Yang M.Y., Wang C.Y., Li P.C., Yu W.C., Hsu Y.F., Wang S.F. (2009). Deposition of low-resistivity gallium-doped zinc oxide films by low-temperature radio-frequency magnetron sputtering. Thin Solid Films.

[19] Yang Z., Look D.C., Liu J.L. (2009). Ga-related photoluminescence lines in Ga-doped ZnO grown by plasma-assisted molecular-beam epitaxy. Appl. Phys. Lett..

[20] Park G.S., Choi W.B., Kim J.M., Choi Y.C., Lee Y.H., Lim C.B. (2000). Structural investigation of gallium oxide (β-Ga_2_O_3_) nanowires grown by arc-discharge. J. Cryst. Growth.

[21] Cheong K.Y., Muti N., Ramanan S.R. (2002). Electrical and optical studies of ZnO:Ga thin films fabricated via the sol-gel technique. Thin Solid Films.

[22] Chang L.W., Yeh J.W., Cheng C.L., Shieu F.S., Shih H.C. (2011). Field emission and optical properties of Ga-doped ZnO nanowires synthesized via thermal evaporation. Appl. Surf. Sci..

[23] Rao T.P., Kumar M.C.S. (2010). Physical properties of Ga-doped ZnO thin films by spray pyrolysis. J. Alloys Compd..

[24] Park S.M., Ikegami T., Ebihara K. (2006). Effects of substrate temperature on the properties of Ga-doped ZnO by pulsed laser deposition. Thin Solid Films.

[25] Chen H., Du Pasquier A., Saraf G., Zhong J., Lu Y. (2008). Dye-sensitized solar cells using ZnO nanotips and Ga-doped ZnO films. Semicond. Sci. Technol..

[26] Le H.Q., Lim S.K., Goh G.K.L., Chua S.J., Ong J.X. (2010). Optical and Electrical Properties of Ga-Doped ZnO Single Crystalline Films Grown on MgAl_2_O_4_ by Low Temperature Hydrothermal Synthesis. J. Electrochem. Soc..

[27] Rana A.S., Ko K., Hong S., Kang M., Kim H.S. (2015). Fabrication and Characterization of ZnO Nanorods on Multiple Substrates. J. Nanosci. Nanotechnol..

[28] Wang H., Baek S., Song J., Lee J., Lim S. (2008). Microstructural and optical characteristics of solution-grown Ga-doped ZnO nanorod arrays. Nanotechnology.

[29] Park G.C., Hwang S.M., Lim J.H., Joo J. (2014). Growth behavior and electrical performance of Ga-doped ZnO nanorod/p-Si heterojunction diodes prepared using a hydrothermal method. Nanoscale.

[30] Rana A.S., Chang S.B., Chae H.U., Kim H.S. (2017). Structural, optical, electrical and morphological properties of different concentration sol-gel ZnO seeds and consanguineous ZnO nanostructured growth dependence on seeds. J. Alloys Compd..

[31] Song J., Lim S. (2007). Effect of Seed Layer on the Growth of ZnO Nanorods. J. Phys. Chem. C.

[32] Barnard A.S., Russo S.P., Snook I.K. (2003). Electronic band gaps of diamond nanowires. Phys. Rev. B.

[33] Degen A., Kosec M. (2000). Effect of pH and impurities on the surface charge of zinc oxide in aqueous solution. J. Eur. Ceram. Soc..

[34] Amin M., Shah N.A., Bhatti A.S., Malik M.A. (2014). Effects of Mg doping on optical and CO gas sensing properties of sensitive ZnO nanobelts. CrystEngComm.

[35] Shanon R.D. (1976). Revised effective ionic radii and systematic studies of interatomic distances in halides and chalcogenides. Acta Crystallogr. Sect. A.

[36] Rana A.S., Lee J.Y., Shahid A., Kim H.S. (2017). Growth Method-Dependent and Defect Density-Oriented Structural, Optical, Conductive, and Physical Properties of Solution-Grown ZnO Nanostructures. Nanomaterials.

[37] Wang Y.G., Lau S.P., Lee H.W., Yu S.F., Tay B.K., Zhang X.H., Tse K.Y., Hng H.H. (2003). Comprehensive study of ZnO films prepared by filtered cathodic vacuum arc at room temperature. J. Appl. Phys..

[38] Rana A.S., Kang M., Kim H.S. (2016). Microwave-assisted Facile and Ultrafast Growth of ZnO Nanostructures and Proposition of Alternative Microwave-assisted Methods to Address Growth Stoppage. Sci. Rep..

[39] Reynolds D.C., Look D.C., Jogai B. (2000). Combined effects of screening and band gap renormalization on the energy of optical transitions in ZnO and GaN. J. Appl. Phys..

[40] Fair R.B. (1979). The effect of strain-induced band-gap narrowing on high concentration phosphorus diffusion in silicon. J. Appl. Phys..

[41] Chen C.W., Chen K.H., Shen C.H., Ganguly A., Chen L.C., Wu J.J., Wen H.I., Pong W.F. (2006). Anomalous blueshift in emission spectra of ZnO nanorods with sizes beyond quantum confinement regime. Appl. Phys. Lett..

[42] Burstein E. (1954). Anomalous Optical Absorption Limit in InSb. Phys. Rev..

[43] Kumar K.D.A., Valanarasu S., Kathalingam A., Ganesh V., Shkir M., AlFaify S. (2017). Effect of solvents on sol-gel spin-coated nanostructured Al-doped ZnO thin films: A film for key optoelectronic applications. Appl. Phys. A.

